# The Value of Pepsinogen in GC Screening: A Systematic Review and Meta-Analysis

**DOI:** 10.1155/2019/7087232

**Published:** 2019-01-21

**Authors:** Ling Liu, Junjie Lang, Yuelong Jin, Yan Chen, Weiwei Chang, Yingshui Yao, Jiegen Yu

**Affiliations:** ^1^School of Public Health, Wannan Medical College, 241002, China; ^2^School of Humanities and Management Science, Wannan Medical College, Wenchang West Road, 22 Wuhu, China

## Abstract

**Background:**

The current gold standard for gastric cancer (GC) screening is pathology or a barium meal followed by X-ray. This is not applicable to a wide range of screening capabilities due to the lack of operability. This article used a meta-analysis to evaluate the value of pepsinogen (PG) screening for GC.

**Methods:**

PubMed, EMbase, the Cochrane Library, CNKI, WanFang, VIP, and CBM databases were systematically searched for published studies that used serum PG to diagnose GC. Articles were searched from January 2003 to January 2018. Two reviewers independently screened the literature according to specified inclusion and exclusion criteria. The data were extracted and evaluated, and the quality of the methodologies evaluated using the QUADAS entry. The meta-analysis (MA) was performed using Meta-DiSc 1.4 software. Stata 12.0 software was used to assess publication bias.

**Results:**

A total of 19 studies were finally included from a total of 169,009 cases. The MA showed a combined sensitivity and specificity of 0.56 (95% CI (0.53–0.59), *P* < 0.01) and 0.71 (95% CI (0.70-0.71), *P* < 0.01), respectively. The combined likelihood ratios were +LR = 2.82 (95% CI (2.06–3.86), *P* < 0.01) and −LR = 0.56 (95% CI (0.45–0.68), *P* < 0.01). The combined DOR was 5.41 (95% CI (3.64~ 8.06), *P* < 0.01), and the area under the SROC curve was 0.7468.

**Conclusions:**

Serum PG provides medium levels of sensitivity and specificity for GC assessment. To be used in a clinical setting, further high-quality research must be performed and verified.

## 1. Introduction

Gastric Cancer (GC) is the fifth most common malignancy worldwide [1]. Although the incidence and mortality of GC have decreased in recent years, it remains a leading cause of cancer-associated death [2]. The high death rates from GC are mainly due to late diagnosis due to the lack of diagnostic criteria [3]. Early GC detection can be performed via endoscopy which is minimally invasive [4]. However, due to the associated pain, high costs, and other factors, gastroscopy is not advised for routine GC screening. Pepsinogen (PG) testing has emerged as a promising alternative. GC mortality can be reduced through noninvasive searches for precancerous lesions, particularly gastric atrophy [5]. In addition, early GC may also be suitable for endoscopic mucosal resection or endoscopic submucosal dissection according to the depth of differentiation and mucosal invasion. Recurrence rates in response to this procedure are low.

In recent years, serum PG detection in high-risk GC populations has been used for primary screening, followed by endoscopy, with relative success. Decreased pepsin levels are associated with an increased risk of GC [6]. Low serum PG-I levels and/or low PGI/II ratios can also predict the long-term risks of death from GC in specific cohorts, highlighting its value as a serum biomarker [7]. Since PG screening occurs in generally asymptomatic or surface-healthy patients in high-risk GC cohorts, its low specificity can increase the number of unnecessary examinations and cause psychological burden to the population. In this regard, PG as a screening indicator of GC has been shown to display variation in sensitivity and specificity.

This meta-analysis (MA) collected nearly fifteen years of Asian and European serum PG screening data to evaluate its accuracy for screening GC. Our objective was to provide evidence for the effectiveness of serum PG to diagnose GC in a clinical setting.

## 2. Data and Methods

### 2.1. Search Strategy

We searched PubMed, EMbase, the Cochrane Library, CNKI, WanFang, VIP, and CBM databases. The relevant professional documents were retrieved manually. The search period was from January 2003 to January 2018. Diagnostic tests of PG for GC were obtained using keywords and search terms including pepsinogen, GC, stomach cancer, stomach neoplasms, and gastric neoplasms.

### 2.2. Patient Criteria

#### 2.2.1. Inclusion Criteria

We included all studies in Chinese and English in which PG (PGI or PGII) was used as a diagnostic test for GC in the last 15 years. All studies had literature that could be extracted as complete tables and used the PGI/PGII ratio (PGR) and/or the PGI levels as an index to evaluate GC, providing definite diagnostic thresholds. In all studies, pathological examination or barium meals followed by X-ray were the gold standard for diagnosis. The outcome measures included sensitivity (Sen), specificity (Spe), positive likelihood ratios (+LR), negative likelihood ratios (−LR), and area under the receiver operating characteristic (SROC) curves (AUC).

#### 2.2.2. Exclusion Criteria

The exclusion criteria included abstracts from meetings, studies with ambiguous measurement indexes, and incomplete or unextractable data. Studies in which the data quality was deemed poor and studies with repeatedly published results were also excluded. Studies were excluded if a complete evaluation was not performed using gold standard tests or if PG was combined with other indicators of GC diagnostic assessments.

### 2.3. Literature Screening and Quality Evaluation

Two reviewers independently screened the manuscripts, extracted data, and performed quality evaluation according to the inclusion and exclusion criteria. Disagreements were discussed or referred to third-party experts for adjudication. Data were extracted from studies (1) that included the first author, study location, and time of publication; (2) that were of sufficient sample size and considered age and gold standard evaluations; (3) that included outcome indicators including true positives (TP), false positives (FP), false negatives (FN), and true negatives (TN); and (4) that included quality evaluations of the key elements.

Quality evaluation of the included studies was assessed using the QUADAS tool for the diagnostic evaluation of systematic reviews. The ratings were divided into “yes,” “no,” and “unclear”: “yes” if the standard was satisfied or “no” if unsatisfied and “unclear” if the information could not be accurately obtained.

### 2.4. Statistical Analysis

Meta-Disc 1.4 software was used for all statistical analyses, through the assessment of the effect of the odds ratio (OR) and 95% CI. For heterogeneity tests, *P* < 0.1 and *I*^2^ > 50% indicated significant heterogeneity based on the ROC curve. *P* values and the Spearman correlation coefficient between the logarithm of the sensitivity and the logarithm of the (1 − specificity) were used to judge the existence of a threshold effect. A “shoulder-arm” distribution of the plan and/or a *P* value <0.05 from the Spearman correlation coefficient suggested a threshold effect. The fit to the SROC curve and area under the curve (AUC) were assessed, or other statistical assessments including the *Q*^∗^ index method were employed. If no threshold effect was observed, we calculated the combined Sen, Spe, +LR, −LR, and DOR and compiled SROC curves and calculated the AUC. Deek's test was used to evaluate publication bias using Stata 12.0 software. Test levels of *α* = 0.05 and *P* < 0.05 were deemed statistically significant.

## 3. Results

### 3.1. Literature Screening

**Figure 1:**
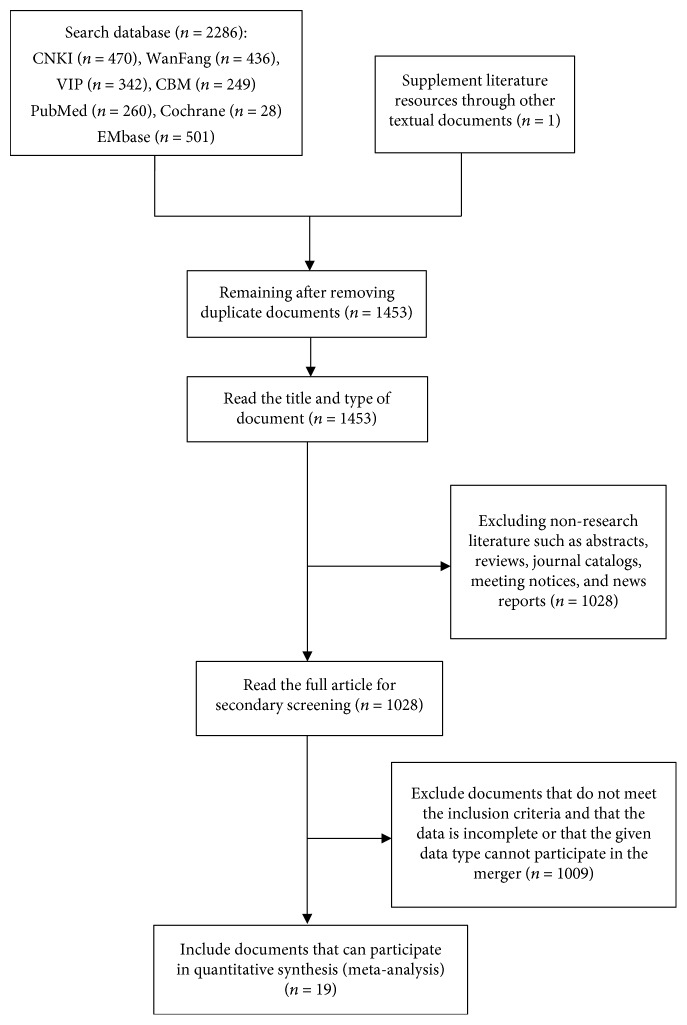
Literature screening process.

A total of 2287 studies were retrieved, 19 of which were included in the final analysis. The gold standard of 16 studies was pathological diagnosis, whilst three studies used a barium meal followed by an X-ray. A total of 169,009 cases received PG screening, of which 67,218 received the gold standard tests (15,566 patients underwent X-ray barium meal tests, and 51,552 patients underwent pathological examination). Screening processes are outlined in Figure 1.

### 3.2. Basic Characteristics and Quality Evaluation

The basic characteristics of the study are included in Table 1, and QUADAS quality evaluations are shown in Table 2.

### 3.3. MA Results

#### 3.3.1. Heterogeneity Analysis

The ROC plane scatter chart did not display a “shoulder arm” appearance, with the Spearman correlation coefficient being 0.457 and *P* = 0.049, indicating no strong correlation between sensitivity and specificity and no threshold effect. The DOR forest map found that the odds ratio of a single study was not distributed in the same line as the combined ratio, indicating the existence of heterogeneity caused by a nonthreshold effect.

#### 3.3.2. Merge Sensitivity and Specificity of PG Screening for GC

**Figure 2:**
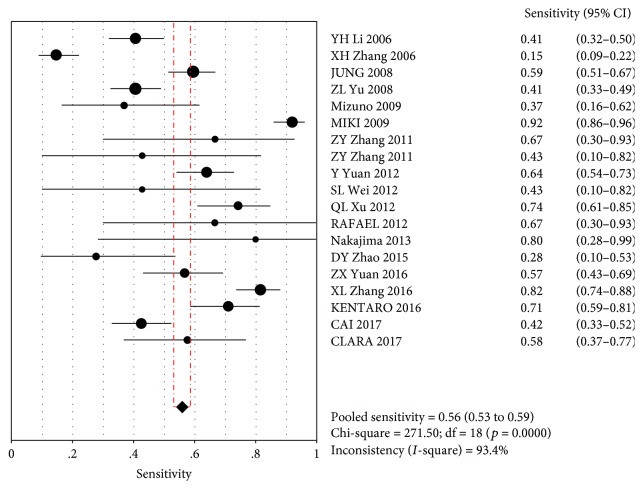
Consolidation sensitivity.

**Figure 3:**
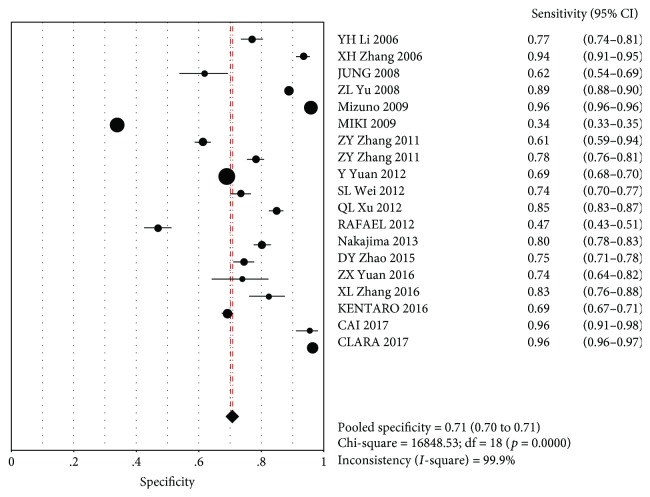
Consolidation specificity.

The MA showed a combined SEN of 0.56 (95% CI (0.53~0.59)) and a combined SPE of 0.71 (95% CI (0.70–0.71)). This indicated that PG did not identify GC in 44% of cases, with misdiagnosis rates of 29% (Figures 2 and 3).

#### 3.3.3. Merge Likelihood Ratio of PG Screening for GC

**Figure 4:**
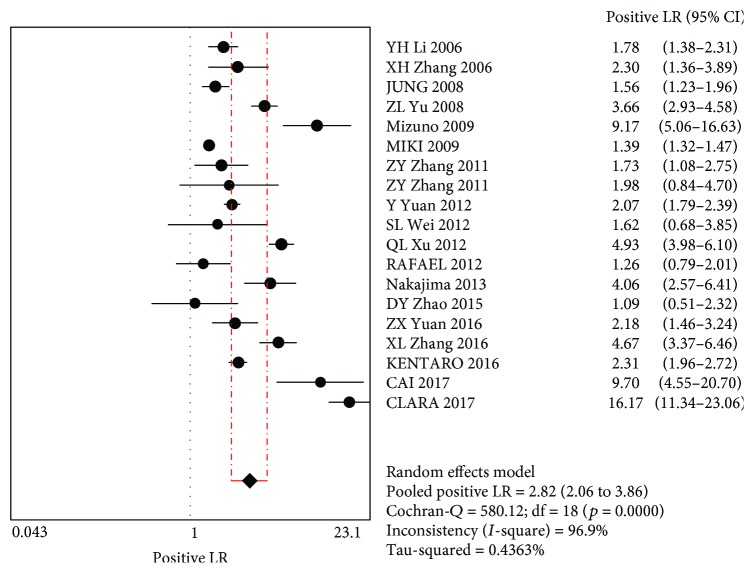
Merging positive likelihood ratios.

**Figure 5:**
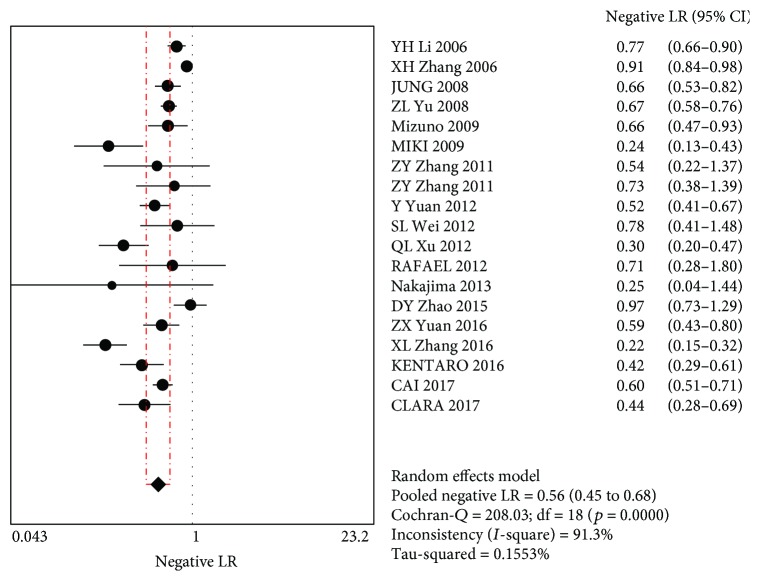
Merging negative likelihood ratios.

The MA showed a combined +LR of 2.82 (95% CI (2.06~3.86)), indicating that the use of PG screening for GC was positive. The combined –LR was 0.56 (95% CI (0.45~0.68)), indicating that when using PG for GC screening, the possibility of missing GC cannot be ruled out (Figures 4 and 5).

#### 3.3.4. Merge DOR of PG Screening for GC

**Figure 6:**
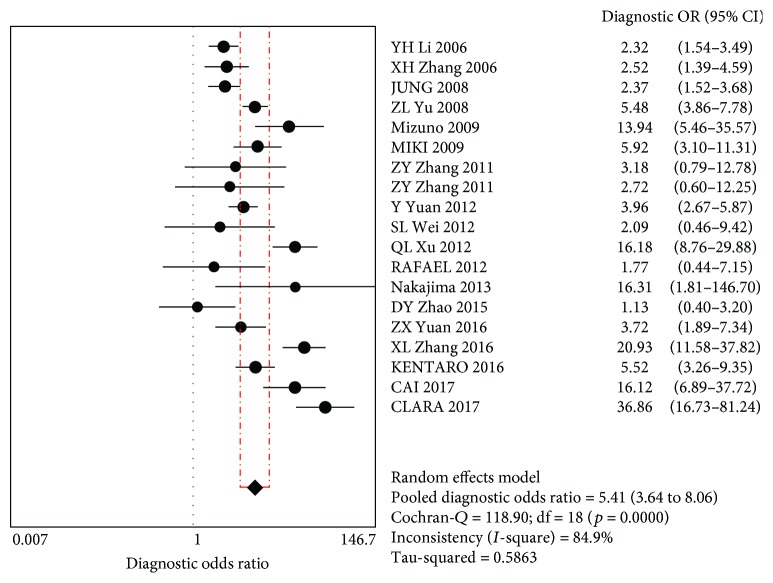
Diagnostic ratios.

DOR forest maps showed that the combined DOR was 5.41 (95% CI (3.64~8.06)), indicating that positive PG screening was 5.41-fold higher than negatively screened patients, suggesting PG has accuracy for GC diagnosis (Figure 6).

#### 3.3.5. SROC and AUC of PG Screening for GC

**Figure 7:**
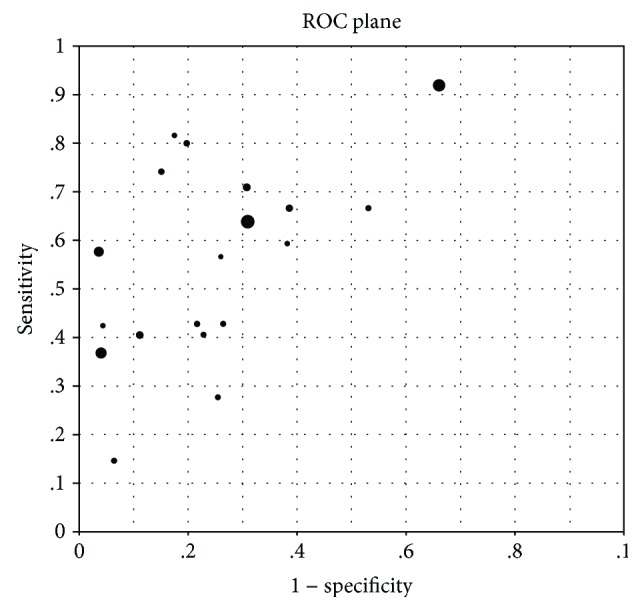
ROC scatter plot.

**Figure 8:**
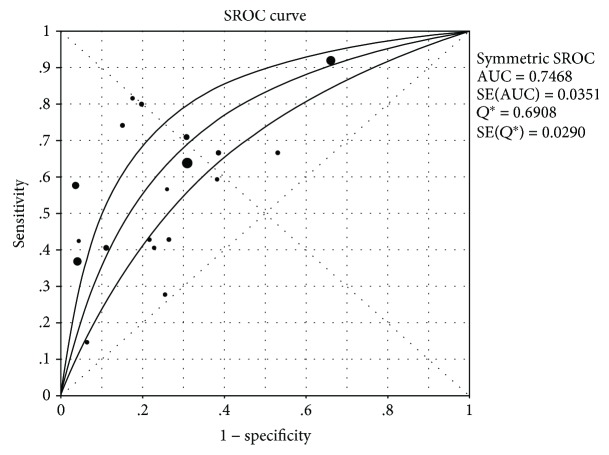
SROC curve.

From the SROC curves, the AUC = 0.7468 and *Q*^∗^ = 0.6908, indicating that PG screening for GC displays only medium efficacy (Figures 7 and 8).

#### 3.3.6. Subgroup Analysis and Sensitivity Analysis

Metaregression was used to analyze the sources of heterogeneity caused by the nonthreshold effect. Subanalysis was conducted based on regional data, publication date, diagnosis method, detection method, and study quality. The results showed a DOR of 3.98 (*I*^2^ = 80.1%, *P* < 0.01) before 2010 and a DOR of 6.24 (*I*^2^ = 84.0%, *P* < 0.01) after 2010. European studies showed a combined DOR of 8.44 (*I*^2^ = 94.0%, *P* < 0.01), whilst the combined DOR in Asia was 5.05 with an *I*^2^ = 82.5% and *P* < 0.01. The combined DOR of the population diagnosed by pathology was 4.96 (*I*^2^ = 86.7%, *P* < 0.01); the combined DOR of the population diagnosed by barium meal and X-ray was 8.54 (*I*^2^ = 44.9%, *P* = 0.163). The combined DOR of the population using ELISA for PG detection was 4.97 (*I*^2^ = 86.6%, *P* < 0.01), whilst the combined DOR of the population using other methods of PG detection was 5.57 (*I*^2^ = 85.2%, *P* < 0.01). The study quality was generally combined with a DOR of 3.72 (*I*^2^ = 84.5%, *P* < 0.01); studies of higher quality were associated with a DOR of 6.91 (*I*^2^ = 86.0%, *P* < 0.01). The study subgroups demonstrated that PG screening of GC was effective to a degree, with medium efficiency (Table 3).

To exclude the impact of low-quality studies on the MA datasets, all studies were analyzed for sensitivity. The results showed that the DOR of PG screening of GC in each group was ≥3 (*P* < 0.05) and the test efficiency was medium, consistent with our data confirming the MA to be of good stability.

## 4. Publication Bias

The results of the funnel plot analysis using Stata 12.0 showed that each circle represented an incorporated study that was approximately symmetrical with respect to the distribution of the central axis (*P* = 0.8). This indicated no publication bias in the study.

## 5. Discussion

Following lung and liver cancer, GC is the third leading cause of global cancer deaths [27]. The high mortality rates of GC are mainly due to undetected symptoms, but when detected early, the 5-year survival rates of GC exceed 90%. Early diagnosis and treatment are key to improving GC therapy, and in this regard, more effective screening and evaluation protocols for GC diagnosis are urgently required [28].

The occurrence and development of GC display regional differences. A significant difference in GC incidence is present between North America and Western Europe, with the highest incidence of GC in East Asia, Eastern Europe, and South America [29]. The incidence of GC and GC associated mortality is highest in Portugal within Western Europe [30]. Despite its importance, many afflicted countries still lack an effective cancer prevention and screening program at the national level. However, in Korea and Japan, the guidelines for screening for GC in high-risk areas were revised in 2015. This included the introduction of organized population-based screening programs [31]. In Japan, the number of deaths associated with GC is approximately 50,000 each year, which has remained consistent over the past three decades [32].

PGI is secreted from the gastric fundus gland, whilst PGII is secreted from the glandular body and the pylorus glands in the antrum and proximal duodenum [33]. The majority of PGs directly enter the stomach cavity, but a small amount also enters the gastric mucosal capillaries and into the bloodstream, which can then be detected in serum. Pepsin is an enzyme that functions specifically in the gastric mucosa. PG is an inactive precursor of pepsin that is mainly synthesized by gastric master cells and cervical mucus cells. Following synthesis, much of the PG is activated into pepsin. Thus, PG can be used to determine gastric mucosal status.

Carcinogenesis of GC is a multistage process in which chronic active gastritis develops leading to atrophic gastritis, intestinal metaplasia, atypical hyperplasia, and eventually cancer development (Correa model) [34]. GC has a multifactorial etiology that is influenced by genetic and environmental predisposing factors. Chronic atrophic gastritis is the leading cause of GC the incidence of which increases with age. Atrophic lesions lead to altered PG secretion from the gastric mucosa [35]. The levels of PG therefore reflect the morphology and functional status of the gastric mucosa. Hence, changes in pepsinogen levels can be used as a serological test for GC and chronic atrophic gastritis [6, 36]. PGI and PGR can directly reflect the number of gastric mucosal glands and cells and indirectly indicate the extent of mucosal atrophy [37].

The International Cancer Research Institute list *Helicobacter pylori* infection as one of the most important carcinogens causing GC [38]. *H. pylori* induces inflammation through gastric mucosal colonization, causing chronic gastritis and mucosal atrophy, which may eventually lead to GC. Large-scale screening for high-risk GC patients through the detection of *H. pylori* has not achieved promising results. The benefits of *H. pylori* screening are related to other baseline GC risks and vary widely amongst populations. An MA of six randomized controlled trials (RCTs) recently conducted in asymptomatic individuals reported that the eradication of *H. pylori* may reduce the risk of GC in the Asian population, but this effect may not be applicable to areas with low GC rates [20]. The impact of large-scale *H. pylori* eradication on the incidence of GC therefore remains unclear. In conditions of limited gastroscopy, endoscopic treatment and other resources may be required to eliminate the burden of GC disease. More simple, reliable, and effective biomarkers are needed to identify those at the highest risk, and as such, PG screening appears to be a more effective choice.

The ROC is a widely accepted method for selecting the optimal cut-off value for a diagnostic test, in addition to assessing its sensitivity and specificity. The AUC represents test effectiveness, with an area > 0.9 indicating a high test efficiency, 0.7–0.9 a medium performance, 0.5–0.7 low efficiency, and 0.5 a chance result [39, 40]. The results of this study showed that the combined sensitivity was 0.56, the combined specificity was 0.71, and the AUC was 0.747, indicating that GC screening using PG was of moderate efficacy, consistent with previous findings [37]. Kang and colleagues [10] demonstrated that the sensitivity and specificity of PG for detecting GC were 59.2% and 61%, respectively, based on a PGR value of ≤3 and 72.4% and 20.2%, respectively, based on a PGI value of ≤70 ng/mL. However, Kitahara and coworkers [41] found that for a PGR ≤ 70 ng/mL, PGR ≤ 4/PGI ≤ 30 ng/mL, and PGR ≤ 3, a higher sensitivity is observed, but the specificity level is poor. When PGI ≤ 70 ng/mL and PGR ≤ 3, the sensitivity and specificity were 84.6% and 73.5%, respectively, which were considered the optimal cut-off point based on the available criteria. Agkoc et al. [1] recorded an optimal cut-off value of PGI ≤ 25 ng/mL and a PGR < 3.0. The positive indicators of PG selected from each study also differed, which are reflected by the known variations of PG screening for GC in different countries and regions. These variations may be related to differences in race, environment, and living habits. Long-term cohort studies in Western countries suggest that PG assessment should be employed for GC screening, which should be repeated every 3 years and further optimized for gender, age, *H. pylori* status, a family history of cancer, and cost [26].

This study had some notable limitations: (1) only Chinese and English studies were searched leading to bias in the study selection. (2) Blinding and randomness of some of the studies were unclear, and the study quality was variable, leading to variations in the obtained data. (3) Due to the inability to obtain age information for all subjects, it was not possible to assess age as a possible confounding factor. (4) Due to the limitations of the included research content, the definition of high-risk groups differed according to regions and detailed experimental methodologies were not reported in detail. Some studies lacked data when classifying the tumor locations/types, meaning the sensitivity and specificity of different types of GC screening may vary. This meta-analysis was based on literature reporting as opposed to direct patient data, also limiting the study.

In summary, we report that PG contributes to the diagnosis of GC displaying moderate diagnostic performance. Although no studies have directly demonstrated that PG screening methods can reduce GC mortality, it does provide a valuable measure to identify high-risk groups who require endoscopy. To provide more scientific and objective references for clinical applications, further research is required using rigorous design, large sample sizes, and multicenter diagnostic assessments. Adopting a unified detection method and strict quality control measures is necessary to reduce bias and to ensure that all research results are of high credibility and strong instructional significance. Following these guidelines can lead to safer, economical, convenient, and accurate methods for screening high-risk groups of GC.

## Figures and Tables

**Table 1:** Basic characteristics of the study.

Reference	Location	Sample size	Age	Gold standard	TP	FP	FN	TN	Critical value	Inspection method
Li et al. [8]	Hebei, China	720	—	Pathology	50	136	73	461	PGR ≤ 6.0	TRFIA
Zhang et al. [9]	Hebei, China	720	—	Pathology	18	38	105	559	PGI < 60 *μ*g/L PGR ≤ 6.0	TRFIA
Kang et al. [10]	Korea	1006	57.6 ± 13.2	Pathology	98	63	67	102	PGR ≤ 3.0	LEI
Yu et al. [11]	Beijing, China	2668	—	Pathology	60	279	88	2241	PGI ≤ 0.7 *μ*g/L PGR ≤ 3.0	ABA
Mizuno et al. [12]	Japan	12,120	15~84	Barium meal	7	486	12	116 15	PGI ≤ 30 ng/mL PGR ≤ 2.0	CT
Miki et al. [13]	Japan	101,892	—	Pathology	115	902 1	10	464 3	PGI ≤ 70 ng/mL PGR ≤ 3.0	RIA
Zhang et al. [14]	Gansu, China	918	>50	Pathology	3	197	4	714	PGI ≤ 70 *μ*g/L PGR ≤ 3.0	ABA
Zhang et al. [15]	Gansu, China	1502	—	Pathology	6	576	3	917	PGI ≤ 70 ng/mL PGR ≤ 7.0	ELISA
Yuan [16]	Liaoning, China	21,338	10~87	Pathology	69	656 0	39	146 70	PGR ≤ 7.0	ELISA
Wei et al. [17]	Hebei, China	753	>35	Pathology	3	197	4	549	PGI ≤ 75 *μ*g/L PGR ≤ 3.5	LEI
Xu et al. [18]	Jiangsu, China	1028	22~91	Pathology	43	146	15	824	PGI ≤ 70 ng/mL PGR ≤ 3.0	LEI
Lomba-Viana et al. [19]	Portugal	13,118	40~79	Pathology	6	268	3	237	PGI ≤ 70 ng/mL PGR ≤ 3.0	ELISA
Nakajima [20]	Japan	1000	—	Barium meal	4	196	1	799	PGI ≤ 70 ng/mL PGR ≤ 3.0	RIA
Zhao et al. [21]	Shanxi, China	725	—	Pathology	5	180	13	527	PGI ≤ 70 *μ*g/L PGR ≤ 3.0	ELISA
Yuan [22]	Shandong, China	160	65.2 ± 4.8	Pathology	34	26	26	74	PGI ≤ 70 ng/mL PGR ≤ 7.0	ELISA
Zhang et al. [23]	Beijing, China	518	13~86	Pathology	102	32	23	151	PGI ≤ 62.5 *μ*g/L PGR ≤ 3.1	CMI
Shikata et al. [24]	Japan	2446	—	Barium meal	49	731	20	164 6	PGI ≤ 59 ng/mL PGR ≤ 3.9	RIA
Juan Cai et al. [25] 2017	Xinjiang, China	464	53.3 ± 13.8	Pathology	45	7	61	153	PGI ≤ 72.78 ng/m L PGR ≤ 4.15	ELISA
Castro et al. [26] 2017	Portugal	5913	40~74	Pathology	15	210	11	567 7	PGI ≤ 70 ng/mL PGR ≤ 3.0	ELISA

**Table 2:** QUADAS quality evaluation.

Inclusion study	(1)	(2)	(3)	(4)	(5)	(6)	(7)	(8)	(9)	(10)	(11)	(12)	(13)	(14)
Li et al. [8]	Y	Y	Y	Y	Y	Y	Y	Y	Y	N	Y	Y	U	U
Zhang et al. [9]	Y	Y	Y	Y	Y	Y	Y	Y	Y	N	Y	Y	U	U
Kang et al. [10]	Y	Y	Y	Y	Y	Y	Y	Y	Y	N	Y	Y	U	U
Zhonglin et al. [11]	Y	Y	Y	Y	Y	Y	Y	Y	Y	N	Y	Y	U	U
Mizuno et al. [12]	Y	Y	Y	Y	Y	Y	Y	Y	N	N	Y	Y	U	U
Miki et al. [13]	Y	Y	Y	Y	Y	Y	Y	Y	Y	N	Y	Y	U	U
Zhang et al. [14]	Y	Y	Y	Y	Y	Y	Y	Y	Y	U	U	Y	U	U
Zhang et al. [15]	Y	Y	Y	Y	Y	Y	Y	Y	Y	U	U	Y	U	U
Yuan [16]	Y	Y	Y	Y	Y	Y	Y	Y	Y	Y	N	Y	U	U
Wei et al. [17]	Y	Y	Y	Y	Y	Y	Y	Y	N	U	U	Y	U	U
Xu et al. [18]	Y	Y	Y	Y	Y	Y	Y	Y	Y	Y	N	Y	U	U
Lomba-Viana et al. [19]	Y	Y	Y	Y	Y	Y	Y	Y	Y	Y	N	Y	U	Y
Nakajima et al. [20]	Y	Y	Y	Y	Y	Y	Y	Y	Y	Y	N	Y	U	U
Zhao et al. [21]	Y	Y	Y	Y	Y	Y	Y	Y	Y	N	Y	Y	U	U
Yuan [22]	Y	Y	Y	Y	Y	Y	Y	Y	Y	N	Y	Y	U	U
Zhang et al. [23]	Y	Y	Y	Y	Y	Y	Y	Y	Y	N	Y	Y	U	U
Shikata et al. [24]	Y	Y	Y	Y	Y	Y	Y	Y	Y	U	U	Y	U	Y
Juan Cai et al. [25]	Y	Y	Y	Y	Y	Y	Y	Y	Y	N	Y	Y	U	U
Castro et al. [26]	Y	Y	Y	Y	Y	Y	Y	Y	Y	Y	N	Y	U	Y

(1) Does the spectrum of cases contain various cases and/or confusing cases? (2) Is the selection criteria for the study object clear? (3) Can the gold standard accurately distinguish sick from disease-free status? (4) Are the intervals between the gold standard and the test to be evaluated short enough to avoid changes in disease conditions? (5) Are all samples or randomly selected samples accepted gold standard tests? (6) Did all cases receive the same gold standard test regardless of the outcome of the trial to be evaluated? (7) Is the gold standard test independent of the test to be evaluated (i.e., the test to be evaluated is not included in the gold standard)? (8) Is the operation of the test to be evaluated described sufficiently clearly and repeatedly? (9) Is the operation of the gold standard test well described and repeatable? (10) Are the results of the test to be evaluated performed without prior knowledge of the gold standard test? (11) Is the interpretation of the outcome of the gold standard test conducted without knowledge of the test results to be evaluated? (12) Is the clinical data available when interpreting the test results and is it consistent with the clinical data available in the actual application? (13) Have you reported any hard-to-interpret/intermediate test results? (14) Have the cases removed from the study been explained?

**Table 3:** Subgroup analysis summary.

Subgroup		DOR	*I* ^2^	*P* value
Date of publication	Before 2010	3.98	80.10%	<0.01
	After 2010	6.24	84.00%	<0.01

Country/region	Europe	8.44	94.00%	<0.01
	Asia	5.05	82.50%	<0.01

Diagnosis method	Pathology	4.96	86.70%	<0.01
	Barium meal	8.54	44.90%	0.163

Detection method	ELISA	4.97	86.60%	<0.01
	Others	5.57	85.20%	<0.01

Document quality	Generally	3.72	84.50%	<0.01
	Higher	6.91	86.00%	<0.01

## Data Availability

The data supporting this meta-analysis are from previously published studies and data sets, which have been cited. The processed data are available in PubMed.
